# Antifungal activity of a novel synthetic polymer M451 against phytopathogens

**DOI:** 10.3389/fmicb.2023.1176428

**Published:** 2023-05-19

**Authors:** Victor Tetz, Kristina Kardava, Konstantin Krasnov, Maria Vecherkovskaya, George Tetz

**Affiliations:** Human Microbiology Institute, New York, NY, United States

**Keywords:** *Ascomycota*, *Oomycota*, *Basidiomycota*, *Fusarium oxysporum*, phytopathogenic fungi, plant disease, M451, antifungal

## Abstract

Phytopathogenic fungi are the predominant causal agents of plant diseases. Available fungicides have substantial disadvantages, such as being insufficiently effective owing to intrinsic tolerance and the spread of antifungal resistance accumulating in plant tissues, posing a global threat to public health. Therefore, finding a new broad-spectrum fungicide is a challenge to protect plants. We studied the potency of a novel antimicrobial agent, M451, a 1,6-diaminohexane derivative, against different phytopathogenic fungi of the *Ascomycota*, *Oomycota*, and *Basidiomycota* phyla. M451 exhibited significant antifungal activity with EC_50_ values from 34–145 μg/mL. The minimal fungicidal concentration against *Fusarium oxysporum* ranged from 4 to 512 μg/mL depending on the exposure times of 5 min to 24 h. M451 has the highest activity and significantly lower exposure times compared to different polyene, azole, and phenylpyrrole antifungals. The conidial germination assay revealed that M451 induced 99 and 97.8% inhibition against *F. oxysporum* within 5 min of exposure to 5,000 and 500 μg/mL, respectively. Germ tube elongation, spore production, and spore germination were also significantly inhibited by M451 at concentrations of ≥50 μg/mL. Based on the broad spectrum of antifungal effects across different plant pathogens, M451 could be a new chemical fungicide for plant disease management.

## 1. Introduction

Phytopathogenic fungi are among the predominant causal agents of plant diseases and can severely interrupt the normal growth of crops, fruits, and vegetables (Blackwell, 2011; Fisher et al., 2012; Doehlemann et al., 2017; Jain et al., 2019). Over 19,000 fungal species, primarily representatives of the *Ascomycota*, *Oomycota*, and *Basidiomycota* phyla, cause various plant infections ranging from persisting in and using living plant tissues to killing the plants to extract nutrients (Doehlemann et al., 2017). Along with direct economic effects due to pre- and post-harvest plant losses, fungal pathogens secrete multiple secondary metabolites, including mycotoxins, which can contaminate agricultural products that harm animal and human health (Gurikar et al., 2022). Moreover, some plant fungi can cause opportunistic infections in humans and other animals, which, although relatively rare, are characterized by broad resistance to antifungal agents (Ma et al., 2013).

Representatives of the *Fusarium* genus are some of the most economically critical fungal phytopathogens, potent mycotoxin producers, and most frequent opportunistic human pathogens (Lazarovits et al., 2014; Doehlemann et al., 2017; Möller and Stukenbrock, 2017). Plant diseases caused by *Fusarium* species are challenging to prevent and control as the infection may be transmitted through the roots in soil or via air or water in above-ground parts (Ma et al., 2013). *Fusarium oxysporum* is one of the top 10 fungal plant pathogens, affecting the yield, quality, and storage life of harvested plants. The economic impact of diseases caused by *Fusarium* determines the relevance of using fungicides in agriculture (McGrath, 2004; Dean et al., 2012; Arie, 2019; Steinberg and Gurr, 2020).

The limited number of existing fungicides and the high resistance to available antifungal compounds contribute to the challenges in controlling fungal plant diseases (Doehlemann et al., 2017). Moreover, many environmental antibiotics, such as azoles or polyenes, used in agriculture are closely related to those used to treat human fungal infections resulting in cross-resistance to the drugs in humans (Lucas et al., 2015; Dalhoff, 2018; Brauer et al., 2019; Matsuzaki et al., 2020; Bastos et al., 2021; Jørgensen and Heick, 2021). Therefore, highly efficient novel antifungal agents not used in human medicine to control agricultural fungal diseases are needed.

Our group previously developed a novel antimicrobial agent, Mul-1867, which possesses a broad spectrum of antimicrobial activity by nonspecifically attacking the fungal cell wall (Tetz et al., 2017; Tetz and Tetz, 2022b). Mul-1867 is effective against *Candida* spp. and *Aspergillus* spp. isolated from patients with fungal lung infections. Mul-1867 was highly effective against resistant clinical isolates, and low minimum fungicidal concentrations were also effective against pre-formed yeast and mold biofilms.

In the present study, we studied the derivative of Mul-1867 developed by our group, TGV-28 (poly-(N-carboxamido-1,6-diaminohexane) particularly N′-aminated) for the first time, which had more potent antifungal activity against different unrelated phytopathogens including *Fusarium* spp., *Blumeria* spp., *Claviceps* spp., *Alternaria* spp., and *Phytophthora* spp. We also studied the potentiation of the antifungal activity of TGV-28 against phytopathogenic fungi by using M451, a is a phosphate salt of TGV-28 and the permeation enhancer 0.01% NaH2PO4. (Dahlgren et al., 2020).

## 2. Materials and methods

### 2.1. Fungal isolates

The *in vitro* antifungal activity was determined against the following plant pathogenic fungi: *F. oxysporum* VT-23, *F. culmorum* VT-45, *F. graminearum* VT-60, *F. sporotrichioides* VT-102, *F. solani* VT-60, *F. verticillioides* VT-125 (all from Human Microbiology Institute collection, NY, United States), *F. proliferatum* KSU 4853 (Kansas State University, KS, United States), *F. dimerum* KSU 14971 (Kansas State University KS, United States), *Blumeria graminis*, *Claviceps purpurea*, *Alternaria alternata*, and *Phytophtora infestans* (all from the Human Microbiology Institute collection). All tested isolates were identified using classic cultivation methods and coupled with 16S ribosomal RNA sequencing for isolate identification and preserved as pure cultures in the collection of the Human Microbiology Institute (New York, NY, United States) (Camele et al., 2021). The fungi were maintained as pure cultures at 4°C and subcultured on potato dextrose agar (PDA; CM0139; Thermo Fisher Scientific Inc., Waltham, MA United States) (Camele et al., 2021).

### 2.2. Tested antifungals

**Figure 1:**
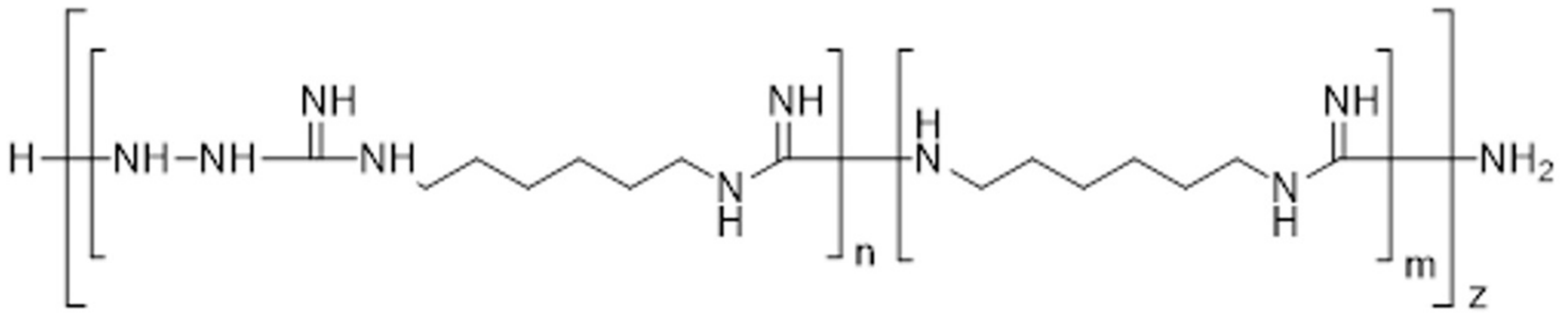
Chemical structure of TGV-28, the active antimicrobial ingredient in M451.

The experimental compound M451 (Human Microbiology Institute) is a phosphate salt of TGV-28 (poly-(N-carboxamido-1,6-diaminohexane) particularly N′-aminated) and the permeation enhancer 0.01% NaH_2_PO_4_ (Figure 1). TGV-28, a derivative of Mul-186, has more polymeric chains than its predecessor, rendering higher antifungal activity (Winiwarter et al., 2007).

We used agriculture-purpose antifungals as positive controls: prothioconazole and tebuconazole (both azole derivatives), natamycin (a polyene derivative), fludioxonil, NaH_2_PO_4_ (all from Sigma–Aldrich, St. Louis, MO, United States), and Maxim® XL (Syngenta Canada Inc., Canada).

### 2.3. Mycelial growth inhibition

Mycelial growth inhibition was evaluated as previously described (Baggio et al., 2014; Chen et al., 2014). For subculturing, varying concentrations of fungicidal agents were added to 90-mm Petri dishes filled with PDA (cooled to ~50°C) before it solidified to obtain M451 0.1%, M451 0.05%, NaH_2_PO_4_ 0.1%, or fludioxonil 0.25% plates. The control plate contained untreated PDA medium. To test the 13 fungal strains, 3 mm diameter mycelium plugs of each fungal strain were cut from the edge of a 5 day-old colony grown on stock PDA plates, transferred to the control and experimental Petri dishes, and incubated at 25°C for 120 h. The diameter of the colonies was measured, and the growth inhibition percentage was calculated.

### 2.4. Spore production

Spore production was determined as previously described (Baggio et al., 2014). To test *F. oxysporum* spore production, 3 mm diameter mycelial plugs were taken from the edge of a five-day-old colony and transferred into the control and test tubes. The control tubes contained 10 mL dH_2_0, 10 μL Tween 20, and 30 μL lactoglycerol as spore germination preventers. The test tubes contained the same mixture of fungicides at various concentrations. The spores were counted using a Malassez counting chamber (Thermo Fisher Scientific, United States) by transferring 0.2 mL from each tube.

### 2.5. Spore germination assay

Spore germination was analyzed using a previously described method (Benslim et al., 2017), in which 1 mL of spore suspension (10^7^ spores/mL) was placed in a series of microtubes, and 20 μL of M451 was added. The tubes were prepared in triplicate and incubated for 24 h at 25°C. After incubation, spore germination inhibition was observed under an AxioStar Plus light microscope (Carl Zeiss, Germany; objective lens: A-Plan 100х/1.25) using a Malassez cell counting chamber. The percentage of non-germinated spores was calculated using Equation 1:


$$\%\mathrm{SNG}=\frac{\mathrm{SNG}}{\mathrm{SG}+\mathrm{SNG}}*100$$

%SNG: percentage of non-germinated spores.

SG: number of germinated spores.

SNG: number of non-germinated spores.

### 2.6. Minimal fungicidal concentration

The minimal fungicidal concentration (MFC) of the tested products was assessed using the standard broth microdilution method, according to the recommendations of the Clinical and Laboratory Standards Institute with RPMI 1640 (Thermo Fisher Scientific, United States) (Clinical and Laboratory Standards Institute, 2008). The standard inoculum for fungal testing was a fungal suspension of 0.5 McFarland. MFC was defined as the lowest concentration of the tested compound resulting in no visual growth after 48 h of incubation at 25°C. After the experiment, the microdilution plates were examined for visible *Fusarium* spp. growth.

### 2.7. Time-kill *in vitro* experiment

We performed a time-kill test *in vitro* to determine the minimum concentration and exposure time required for the tested compounds to kill the fungi. We assessed the activity of serially diluted compounds against *F. oxysporum* as described by Ernst et al. (1998). All fungal cultures were grown for 120 h as liquid cultures, and 150 μL of the inoculum (5 × 10^5^ colony-forming units (CFU)/mL) for each strain was transferred to 96-well microtiter plates (Sarstedt, Numbrecht, Germany). The plates were incubated for 120 h at 25°C. Then, 50 μL of the tested compounds at different concentrations were added for 5 min, 30 min, 1 h, 3 h, 6 h, and 24 h. Untreated probes were used as negative controls. After exposure, the fungal suspension was centrifuged at 4000 *× g* for 15 min and washed with deionized water. Centrifugation and washing were repeated twice. The probes were diluted in 11 mL phosphate-buffered saline, and the total CFU number was determined through serial dilution and plating on PDA. All assays included at least two replicates and were repeated in three independent experiments.

### 2.8. Germ tube elongation assay

The germ tube elongation was performed following a previously reported procedure (Chen et al., 2014). To test the inhibition of germ tube elongation, we prepared a spore suspension of *F. oxysporum* (10^7^ spores/mL). One hundred microliters of this suspension were placed on control and experimental plates supplemented with M451 at various concentrations. The plates were incubated for 10 h at room temperature. Germ tube length was measured for 100 germinated spores at 400× magnification (Axiostar plus (Carl Zeiss, Germany)) using Fiji software (Schindelin et al., 2012).

### 2.9. Conidial germination assay

The conidial germination assay was conducted as previously reported (Oukhouia et al., 2017). M451 (measured for TGV-28) and fludioxonil were diluted in 0.9% NaCl, mixed with 0.2% agar, and added to 5 day-old *F. oxysporum* conidia suspension (10^6^ conidia/mL) to a final concentration of 5,000, 500, 100, 50, 5, 1, or 0.5 μg/mL. After 1, 3, 6, and 24 h of incubation at 25°C, the number of germinated conidia was determined using the Malassez counting chamber in 10 fields with standard techniques (Niemann and Baayen, 1989).

### 2.10. Statistical analyses

All data were analyzed using GraphPad Prism version 9.3.1. Two-way analysis of variance (ANOVA) was used for multiple comparisons at a 95% confidence level (*p* < 0.05). Data are presented as the mean ± standard deviation (SD) with three independent replicates. All experiments were performed in triplicate.

## 3. Results

### 3.1. Initial antifungal screening assay

We tested TGV-28 and NaH_2_PO_4_, alone and in combination (M451), against different species from the *Ascomycota*, *Oomycota*, and *Basidiomycota* phyla. TGV-28 was highly effective against all strains, with the MFC 64.3–96.3 μg/mL (Table 1). As expected, NaH_2_PO_4_ at a 100 μg/mL concentration did not exhibit antifungal activity (data not shown). Surprisingly, the TGV-28 and NaH_2_PO_4_ combination increased the antifungal activity of TGV-28 against *F. oxysporum* by 49% (*p* < 0.05). Therefore, in subsequent experiments, we used a complex of TGV-28 and NaH_2_PO_4_ named M451.

**Table 1:** Antifungal activity of tested compounds.

Fungi	Minimal fungicidal concentration (μg/mL)	
	TGV-28	M451
*Fusarium culmorum*	1.3 ± 0.6	1.3 ± 0.6
*Fusarium graminearum*	0.2 ± 0.3	0.2 ± 0.3
*Fusarium sporotrichioides*	0.3 ± 0.3	0.3 ± 0.3
*Fusarium oxysporum*	5.3 ± 2.3	2.7 ± 1.2
*Fusarium solani*	2.7 ± 1.2	2.7 ± 1.2
*Fusarium dimerum*	3.3 ± 1.2	3.3 ± 1.2
*Fusarium proliferatum*	1.3 ± 0.6	1.3 ± 0.6
*Fusarium verticillioides*	0.8 ± 0.3	0.8 ± 0.3
*Erysiphe graminis*	1.3 ± 0.6	1.3 ± 0.6
*Clavicens purpurea*	0.2 ± 0.3	1.3 ± 0.6
*Alternaria alternata*	1.7 ± 0.6	1.7 ± 0.6
*Phytophthora infestans*	0.3 ± 0.3	0.2 ± 0.3
*Rhizoctonia solani*	0.3 ± 0.3	0.3 ± 0.3

### 3.2. Antifungal activity of M451 against plant pathogens

M451 exhibited significant activity against all tested plant-pathogenic fungal isolates and inhibited the *in vitro* mycelial growth of all *Ascomycota*, *Oomycota*, and *Basidiomycota* strains (Table 2), with an EC_50_ of 34–145 μg/mL and a high correlation coefficient. The EC_50_ of M451 against *Fusarium* spp. (phylum *Ascomycota*) varied from 66 μg/mL to 145 μg/mL. The activity against other non-*Fusarium* representatives of the *Ascomycota* phylum was higher, with EC_50_ values ranging from 34 to 52 μg/mL. The EC_50_ values for *Phytophtora infestans* (phylum *Oomycota*) and *Rhizoctonia solani* (phylum *Basidiomycota*) were 58 and 53 g/mL, respectively.

**Table 2:** *In vitro* inhibition of plant pathogens by M451.

Phylum	Species	Correlation coefficient*	EC_50_ (μg/mL)
*Ascomycota*	*F. culmorum*	0.977	66
	*F. graminearum*	0.811	67
	*F. sporotrichioides*	0.820	78
	*F. oxysporum*	0.924	85
	*F. solani*	0.849	87
	*F. dimerum*	0.983	145
	*F. proliferatum*	0.992	68
	*F. verticillioides*	0.995	76
	*Erysiphe graminis*	0.930	52
	*Clavicens purpurea*	0.854	43
	*Alternaria alternata*	0.975	34
*Oomycota*	*Phytophthora infestans*	0.977	58
*Basidiomycota*	*Rhizoctonia solani*	0.976	53

*Correlation between the probability value transformed by the percentage of inhibition relative to the control (without fungicide) and the concentration (log-transformed) of the fungicide.

**Figure 2:**
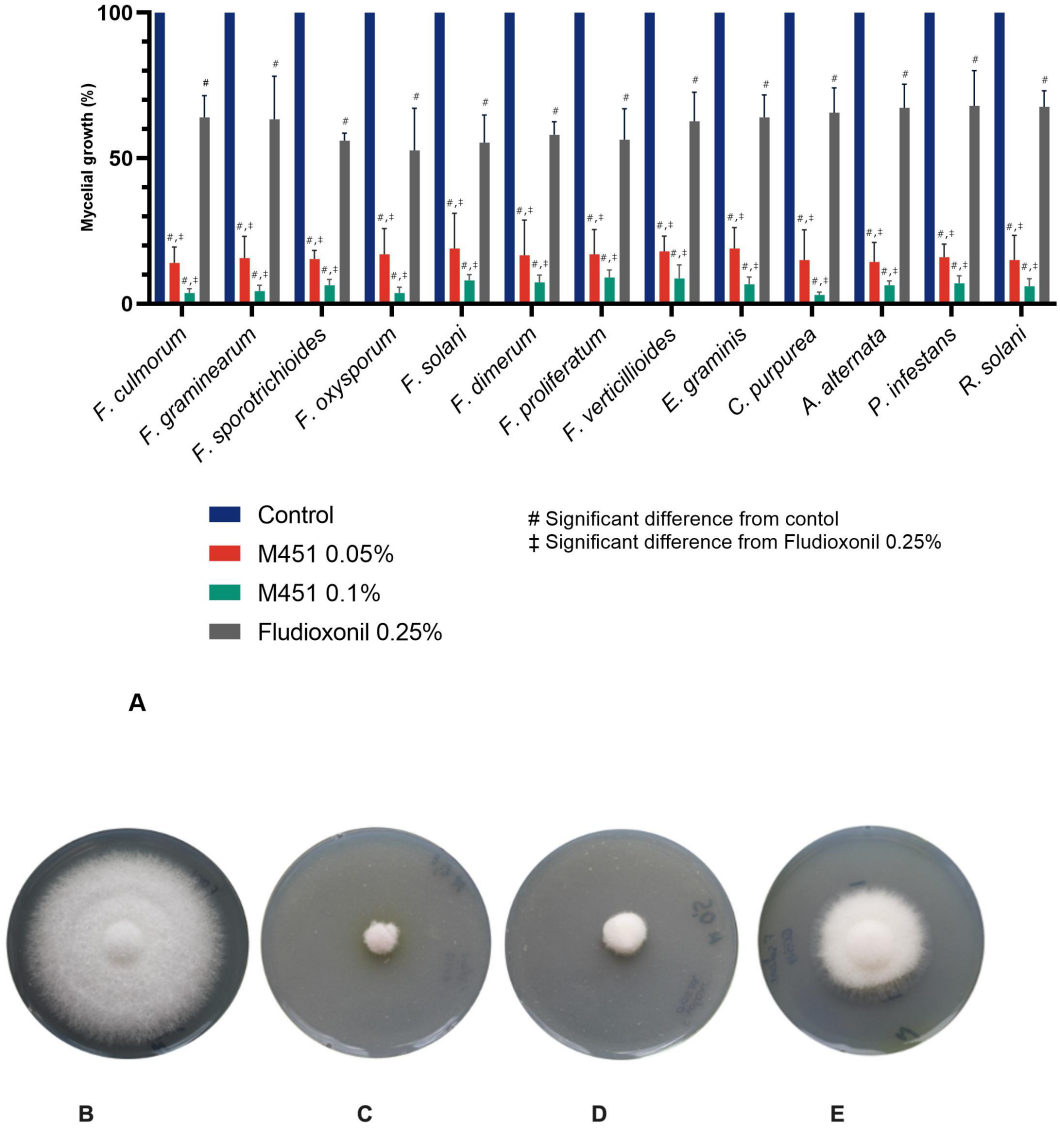
Effect of tested compounds on fungal growth. **(A)** Mycelial growth inhibition of plant phytopathogens. **(B**–**E)** Antifungal activity against *F. oxysporum*. Representative images from three independent experiments of fungi cultivated for five days at 25°C after treatment with the tested compounds. **(B)** Negative control, **(C)** M451 0.1%, **(D)** M451 0.05%, **(E)** Fludioxonil 0.25%. #*p* < 0.05 compared to the control, ‡*p* < 0.05 compared to fludioxonil.

We then compared the antifungal activity of M451 and fludioxonil against the same fungal isolates. M451 revealed dose-dependent activity: 0.1% M451 displayed 91–97% mycelial growth inhibition, and 0.05% M451 resulted in a slightly lower decrease in mycelial growth with 82–86.7% inhibition among different fungal pathogens (Figure 2A). Notably, M451 at a concentration of 0.05 and 0.1% was more effective against all tested fungi than fludioxonil (the active ingredient in Maxim XL), which, when used at a fixed concentration of 0.25% according to the manufacturer’s recommendations, inhibited mycelial growth by only 32–47% (*p* < 0.05 for the control, M451 0.1%, and M451 0.05%). Representative images of the mycelial growth inhibition of *F. oxysporum* by M451 are shown in Figures 2A–E.

### 3.3. The M451 MFC and different antibiotics against *Fusarium oxysporum*

Table 3 shows the MFCs of M451 and antifungal antibiotics used in agriculture representing polyene and azole classes and stand-alone Maxim XL (titrated depending on the fludioxonil concentration) against *F. oxysporum*. The time-kill study against planktonic fungi demonstrated a higher antifungal activity of M451 at all tested time points. At the end of 24 h of exposure, the antifungal activity of M451 was 5–50 times higher than that of other antifungal agents. Notably, M451 was the only tested antifungal agent that showed antifungal activity from 5 min of exposure.

**Table 3:** The MFCs of M451 and antifungal antibiotics against *F. oxysporum.*

Antifungal agent	MFC at different incubation times (μg/mL)					
	5 min	30 min	1 h	3 h	6 h	24 h
M451	512	64	32	16	16	4
Fludioxonil	>1,024	>1,024	512	256	256	256
Prothioconazole	>1,024	>1,024	128	128	32	16
Tebuconazole	>1,024	>1,024	128	32	32	16
Natamycin	>1,024	>1,024	64	64	32	32

### 3.4. Effect of M451 on conidial germination

**Figure 3:**
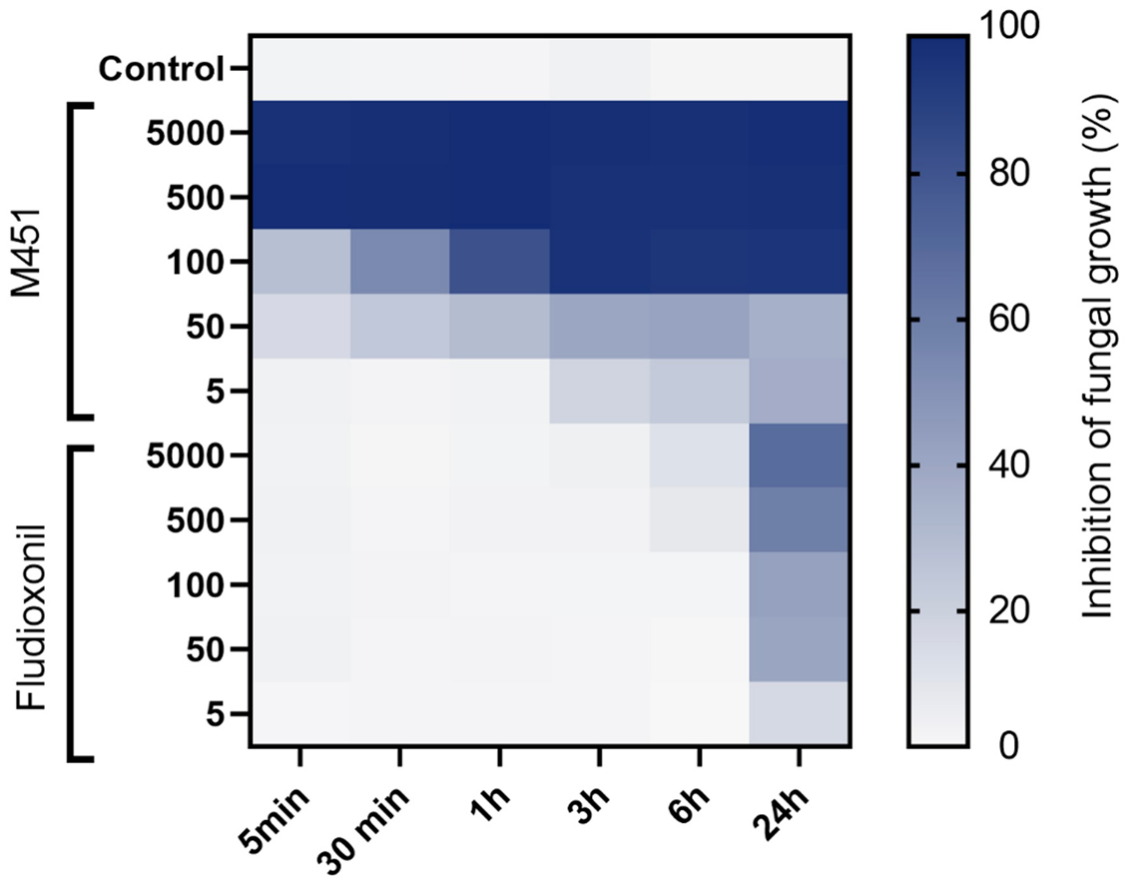
Heatmap of the effect of different M451 and fludioxonil concentrations on *F. oxysporum* conidia. Growth inhibition is represented by a color scale from white (minimal) to blue (maximum). The data are representative of three independent experiments.

Next, we studied the effect of different M451 concentrations corresponding to 5,000–5 μg/mL TGV-28 compared to Maxim XL on *F. oxysporum* conidial germination (Figure 3). M451 treatment at 5000 and 500 μg/mL inhibited conidial germination by 99 and 97.8%, respectively, after 5 min of exposure. We could see the dose- and time-dependent effects of M451 at lower concentrations. Thirty minutes of incubation with M451 at 100 μg/mL resulted in 53.8% inhibition; increasing the incubation time to 3 h resulted in 94.4% inhibition. Under the same conditions, Maxim XL had a much lower level of anti-*Fusarium* activity, which at a 5,000 μg/mL concentration only reduced germination by 3.6 and 67.8% after 5 min and 24 h, respectively.

### 3.5. Effect of M451 on different developmental stages of *Fusarium oxysporum*

**Figure 4:**
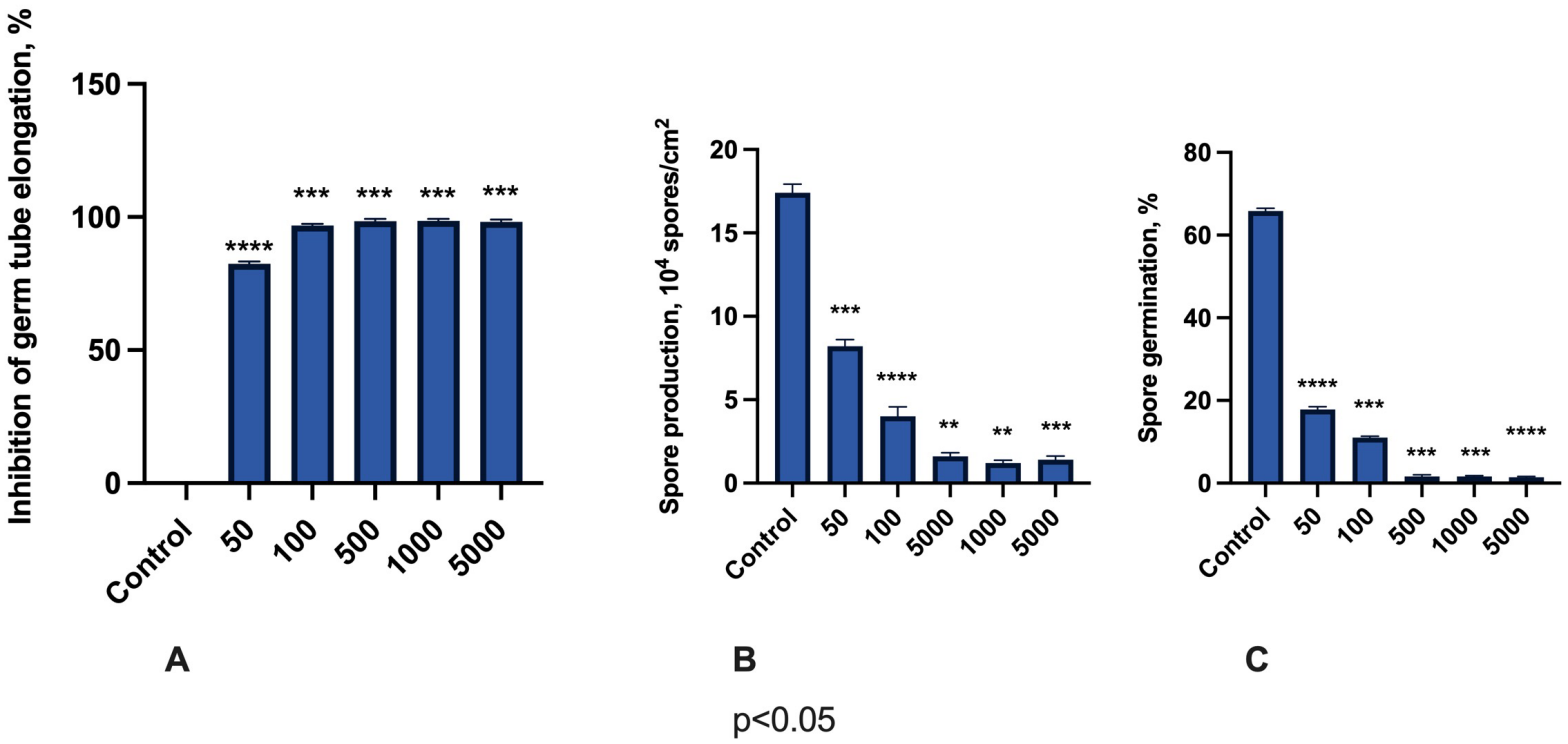
Antifungal effects of M451 on *F. oxysporum*. The bars represent the effects of M541 at concentrations from 50 to 5,000 μg/mL on **(A)** germ tube elongation, **(B)** spore production, and **(C)** spore germination. **p < 0.01, ***p < 0.001, ****p < 0.0001 compared to the control. Compiled data are from three independent experiments.

Finally, we determined the *in vitro* effects of M451 on *F. oxysporum* germ tube elongation, spore production, and germination (Figure 4). Germ tube elongation was highly sensitive to M451 treatment, with >80% inhibition, even when M451 was applied at a sub-inhibitory concentration of 50 μg/mL and over 95% inhibition starting from 100 μg/mL (Figure 4A). Notably, M451 significantly inhibited spore production at all tested concentrations. Even at the lowest tested concentration of 50 μg/mL, M451 inhibited spore production by over 2-fold. This inhibition might be explained by the quick action of M451 when the compound kills fungi faster than the sporulation process starts (Figure 4B). Finally, M451 had a dose-dependent inhibitory effect on *Fusarium* spore germination (Figure 4C). Thus, M451 at 50 and 100 μg/mL reduced spore germination from 73 to 83% compared to the control, respectively, and from concentrations of 500 μg/mL, M451 almost completely inhibited this process.

## 4. Discussion

Plant fungal diseases caused by *Ascomycota*, *Oomycota*, and *Basidiomycota* spp. contributes to severe crop losses and a negative economic effect on essential agricultural products (Gisi and Cohen, 1996; Tudzynski and Scheffer, 2004; Haverkort et al., 2016). The primary difficulties in preventing and treating agricultural fungal diseases are the resistance of phytopathogenic fungi to existing fungicides, tolerant spore development, and insufficient studies on fungal regulation strategies (Gisi and Cohen, 1996; Avenot and Michailides, 2007; Al-Hatmi et al., 2019; Meyers et al., 2019; Jindo et al., 2021; Tetz and Tetz, 2022a,b).

Moreover, developing fungus-specific targets is complicated because fungal and mammalian cells share many of these targets. Therefore, only a few classes of antifungal agents are available, and some are used in agriculture and human health. This everyday use of antifungal agents causes the spread of antibiotic resistance in human medicine in several ways, varying from direct infection with resistant bacteria to the transfer of antibiotic resistance genes from plant to human pathogens (Chang et al., 2015). Therefore, identifying novel antifungal agents that can be used only in agriculture and not in humans is crucial for agriculture and human health.

The novel fungicide candidate TGV-28, a derivative of the broad-spectrum antimicrobial agent Mul-1867, exhibits high antifungal activity against several emergent plant pathogens, including representative *Fusarium* spp., *Erysiphe* spp., *Phytophthora* spp., and *Rhizoctonia* spp. Moreover, its antimicrobial activity against specific fungal plant pathogens was potentiated by the permeation enhancer NaH_2_PO_4_. Therefore, we also studied a complex of TGV-28 and NaH_2_PO_4_ named M451. However, the detailed mechanisms underlying this selective potentiation against only a particular type of fungi (*F. oxysporum*) are a subject for future research and are outside the scope of this study. M451 exhibited significant antifungal activity against *Fusarium* spp. as a seed dressing for wheat seeds, which exceeded the commercially used fungicide Maxim XL against both seed- and soil-borne *F. oxysporum* infections (Kardava et al., 2023).

We were particularly interested in evaluating the antifungal effect of M451 against *F. oxysporum* owing to its importance as a devastating plant pathogen (Ma et al., 2013; De Corato et al., 2020). We compared the activity of M451 with that of different agriculture-purpose antifungal agents, including azole and polyene derivatives and fludioxonil. M451 had lower MFCs at all measured time points and could inhibit the fungal growth even at concentrations substantially lower than the anticipated M451 concentrations (0.05–0.1%); therefore, we envision that M451 can be used in real-world agricultural settings. Moreover, unlike other tested antifungal agents, M451-induced rapid killing of *F. oxysporum* after 5 min of exposure. The fast-acting antifungal effect was expected because TGV-28 and its predecessor Mul-1867 exhibit a topical mechanism of antifungal action without metabolizing it (Tetz et al., 2017). One of the pathways through which fungi overcome antibiotic challenges is to develop highly resistant spores. The results confirmed that M451 prevents fungal survival by negatively affecting sporulation even after a short exposure time.

We compared the activity of M451 and fludioxonil against *F. oxysporum* conidia, as conidial stress resistance is higher than that of vegetative fungal cells (Wyatt et al., 2013). Whereas M451 exhibited a high antifungal activity from 5 min of exposure, fludioxonil required considerably more time to show antifungal activity. At the end of the observation period, fludioxonil could not inhibit fungal growth even at the highest concentration tested, which exceeded two-fold that recommended by the manufacturer. This finding was most likely due to the experimental settings of the conidial germination assay, which enabled fungi to escape from the antifungal effects by asexual sporulation. These results are consistent with recently published data that fludioxonil has limited activity in field studies owing to limited activity towards conidia and sexual spores, although it is broadly used in agriculture to treat and prevent *Fusarium* infection (Masiello et al., 2019).

As fungi are present in the outer environment at different life cycle stages in real-world conditions, we also compared the effect of M451 and fludioxonil on germ tube elongation, spore production, and spore germination. This experiment was conducted considering that *F. oxysporum* overcomes the killing effect of antifungal agents by forming highly tolerant spores. M451 exhibited a high efficacy against *F. oxysporum* at all developmental stages, inhibiting *F. oxysporum* spore germination and even possessing sporicidal activity. This class of compounds does not have significant cytotoxicity; therefore, the observed sporicidal effect could not be addressed owing to corrosive effects and is planned to be studied in the future (Tetz et al., 2017; Tetz and Tetz, 2022b). In conclusion, this study revealed high *in vitro* antifungal activities of M451 against difficult-to-treat plant pathogens. Fungi at different developmental stages frequently characterize real-life plant fungal infections. Therefore, one of the unique M451 features was that it inhibited vegetative proliferation and conidial and spore germination. Finally, M451 represents a novel class of chemical compounds unrelated to antifungal antibiotics used to treat human diseases. M451 does not share structural characteristics with azoles of polyene antibiotics; therefore, it is unlikely to contribute to the developing antibiotic resistance frequently seen across antifungals used in medicine and agriculture (Verweij et al., 2022). Further development of M451 can be done following the local regulatory authorities and include relevant *in vivo* studies.

## Data availability statement

The datasets presented in this study can be found in online repositories. The names of the repository/repositories and accession number(s) can be found at: https://www.biorxiv.org/content/10.1101/2023.02.04.525039v1.

## Author contributions

VT, KKa, and GT conceived and supervised the research and edited and helped to draft the final manuscript. KKa and MV conducted experiments. KKa and KKr analyzed the data and wrote the manuscript. All authors contributed to the article and approved the submitted version.

## Conflict of interest

The authors declare that the research was conducted without any commercial or financial relationships that could be construed as a potential conflict of interest.

## Publisher’s note

All claims expressed in this article are solely those of the authors and do not necessarily represent those of their affiliated organizations, or those of the publisher, the editors and the reviewers. Any product that may be evaluated in this article, or claim that may be made by its manufacturer, is not guaranteed or endorsed by the publisher.
